# EBV induced Evans syndrome in a patient of SLE related MAS: A case report

**DOI:** 10.1097/MD.0000000000050335

**Published:** 2026-08-21

**Authors:** WeiBin Li, Yini Chen, Ya Chen, Meng Zhao, Shenghang Zhang

**Affiliations:** a Department of Clinical Laboratory Medicine, 900th Hospital of PLA Joint Logistic Support Force, Fuzhou, Fujian, China; b Department of Clinical Laboratory Medicine, Fuzong Clinical Medical College of Fujian Medical University, Fuzhou, Fujian, China; c Department of Clinical Laboratory Medicine, Fuzong Teaching Hospital of Fujian University of Traditional Chinese Medicine (900th Hospital), Fuzhou, Fujian, China.

**Keywords:** Evans syndrome, hemophagocytic syndrome, macrophage activation syndrome, systemic lupus erythematosus

## Abstract

**Rationale:**

Epstein-Barr virus (EBV) induced Evans syndrome in systemic lupus erythematosus (SLE) related macrophage activation syndrome (MAS) is rare with the incidence rate of 0.9% to 9% and the mortality rate of about 4% to 19%, which are misdiagnosed as hemophagocytic lymphohistiocytosis.

**Patient concerns:**

A 15-years old female was admitted due to abdominal pain and fever. Physical examination showed butterfly-shaped erythema on her face and scattered lymph nodes of bilateral neck with a diameter of 1.5 cm × 1.0 cm. Pharyngeal mucosal congestion, bilateral tonsil swelling, and palpable 1 cm below the liver rib and no palpable spleen were observed. Medical history revealed recurrent facial erythema after puberty, which is aggravated by exposure to sunlight.

**Diagnoses:**

Based on the results of complete blood cell count, biochemical and immunologic examination and bone marrow cytology, EBV-induced Evans syndrome of SLE-related MAS was reached.

**Interventions:**

Ceftriaxone, oseltamivir, methylprednisolone, omeprazole, cyclosporine, and high-dose immunoglobulin were administrated and after 20 days the patient was discharged and regularly followed up.

**Outcomes:**

After 20 days of administration, the patient was significantly improved and discharged.

**Lessons:**

Disease history, laboratory tests, and differential diagnosis contribute to the confirmation of EBV-induced Evans syndrome in SLE-related MAS.

## 1. Introduction

Hemophagocytic lymphohistiocytosis (HLH), also known as hemophagocytic syndrome, is an inflammatory response syndrome caused by excessive activation and proliferation of cytotoxic T lymphocytes, natural killer cells (NK), and macrophages, leading to multiple organ dysfunction which is severe with high mortality rate.^[1]^ It is characterized by fever, decreased whole blood cells, hepatosplenomegaly, and phagocytosis of macrophages. HLH can be divided into primary and acquired types. Primary HLH is mainly found in children and is an autosomal recessive or X-linked genetic disease caused by a genetic defect that leads to cytotoxic functional defects. Acquired HLH is mostly found in adults, often secondary to viral or bacterial infection, malignant tumors, autoimmune diseases. HLH secondary to rheumatic immune diseases is also called macrophage activation syndrome (MAS), usually associated with adult Still disease and systemic juvenile idiopathic arthritis, systemic lupus erythematosus (SLE) secondary MAS is relatively rare, and the incidence rate is 0.9% to 9%,^[2]^ the mortality rate of MAS secondary to SLE is about 4% to 19%.^[3]^ Here we reported a case of Epstein-Barr virus (EBV) induced Evans syndrome in SLE related MAS.

## 2. Case report

**Figure 1.**
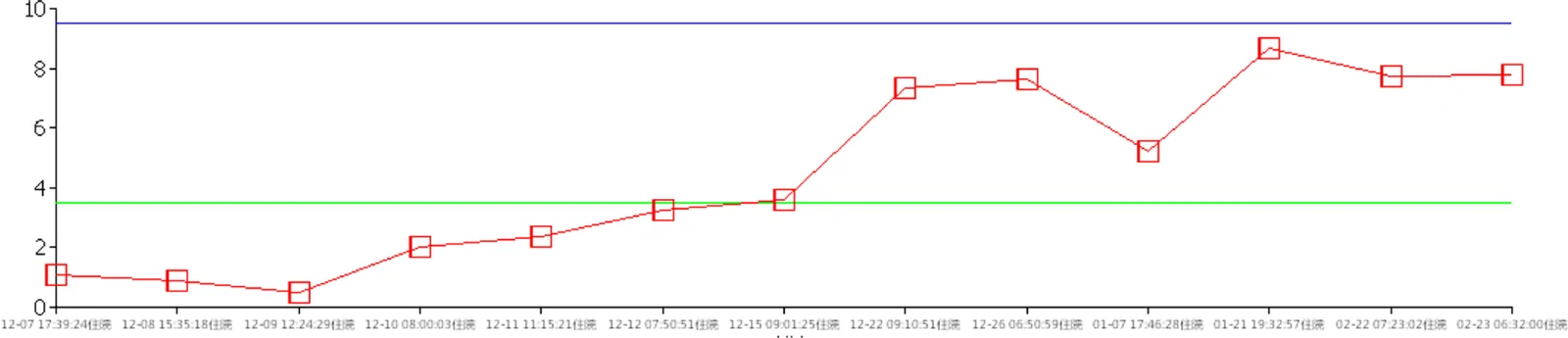
The result of the white blood cell count showed a gradually increasing tendency. When admitted, his complete blood cell count revealed a white blood cell count of 1.13 × 10^9^/L, a neutrophil percentage of 54.9%, and a lymphocyte percentage of 37.8%. On December 8th and 9th, his white blood cells decreased to 0.90 × 10^9^/L and 0.48 × 10^9^/L. From December 10th, his white blood cell count began to gradually increase. *X*-axis: daytime; *Y*-axis: white blood cell count.

**Figure 2.**
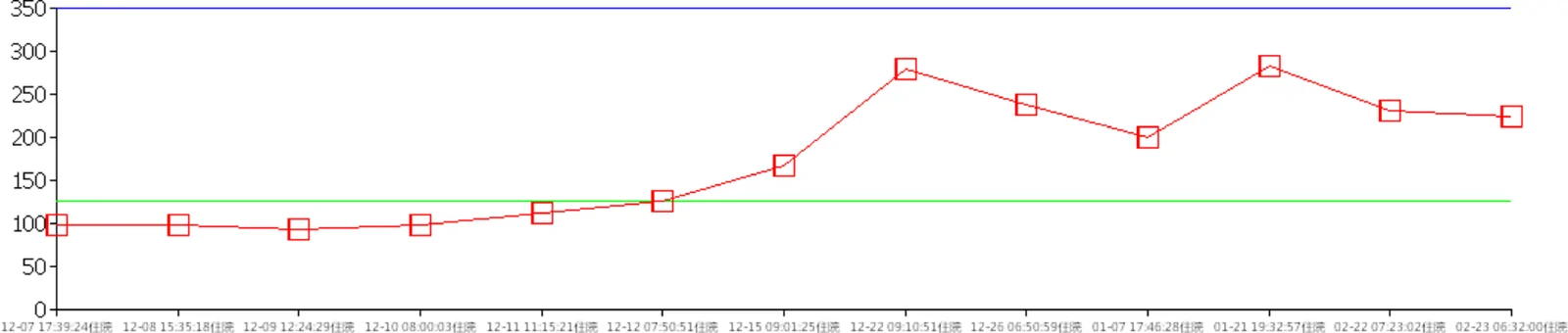
The result of the platelet count showed a gradually increased tendency.When admitted, his complete blood cell count revealed a platelet count of 95.0 × 10^9^/L. From December 15th, his platelet count began to gradually increase. *X*-axis: daytime, *Y*-axis: platelet count.

A 15 years old female was admitted to our hospital on December 7, 2025 due to abdominal pain for 10 days and fever for 4 days. Physical examination showed butterfly-shaped erythema on her face and scattered lymph nodes in the bilateral neck with a diameter of 1.5 cm × 1.0 cm. Pharyngeal mucosal congestion, bilateral tonsil swelling, palpable 1 cm below the liver rib, and no palpable spleen were observed. Medical history revealed recurrent facial erythema after puberty, which is aggravated by exposure to sunlight. Complete blood cell count revealed a white blood cell count of 1.13 × 10^9^/L, neutrophil percentage of 54.9%, lymphocyte percentage of 37.8%, red blood cell count of 4.01 × 10^12^/L, hemoglobin measurement of 118.0 g/L, platelet count of 95.0 × 10^9^/L, reticulocyte percentage of 3.36%, reticulocyte count of 0.1232 × 10^12^/L, and an immature reticulocyte ratio of 16.4%. Complete blood count showed a gradual upward trend after treatment (see Figures 1 and 2). Coombs test showed that the immunoglobulin G (IgG) type was negative and the C3 type was 1:64 positive, and glucose-6-phosphate dehydrogenase activity was normal.

Urea 3.6 mmol/L, creatinine 55.0 μmol/L, total protein 65.6 g/L, albumin 38.6 g/L, globulin 28.5 g/L, alanine aminotransferase 39.0 U/L, total bilirubin 20.03 μmol/L, direct bilirubin 8.88 μmol/L, indirect bilirubin 11.15 μmol/L, creatine kinase isoenzyme 15.0 U/L, aspartate aminotransferase 79.0 U/L, lactate dehydrogenase 1374 U/L, triglycerides 0.54 mmol/L, potassium 4.0 mmol/L, sodium 139.7 mmol/L, calcium 2.49 mmol/L, serum amylase 173.4 U/L, serum lipase 74.6 U/L, and urine amylase 425.9 U/L. Urinary calcium was 7.48 mmol/L, urinary creatinine 8853.2 μmol/L, microalbumin 3.15 mg/L, urinary microalbumin/creatinine ratio 0.36 mg/mmol, and urinary calcium/creatinine 0.84; urine α 1-microglobulin was 46.36 mg/L, urine β 2-MG was 4.16 mg/L; 24-hour urinary protein quantification: protein 0.203 g/L, urine output 1230 mL/24 hours, and 24-hour protein quantification 0.250 g/24 hours. After treatment, indirect bilirubin showed a gradually increasing trend, while lactate dehydrogenase, alanine aminotransferase, and aspartate aminotransferase showed a gradually decreasing trend. Biochemical examination showed serum amylase of 173.4 U/L and 24-hour urinary protein quantification of 0.250 g/24 hours. After treatment, lactate dehydrogenase, alanine aminotransferase, and aspartate aminotransferase showed a gradually decreasing trend.

**Figure 3.**
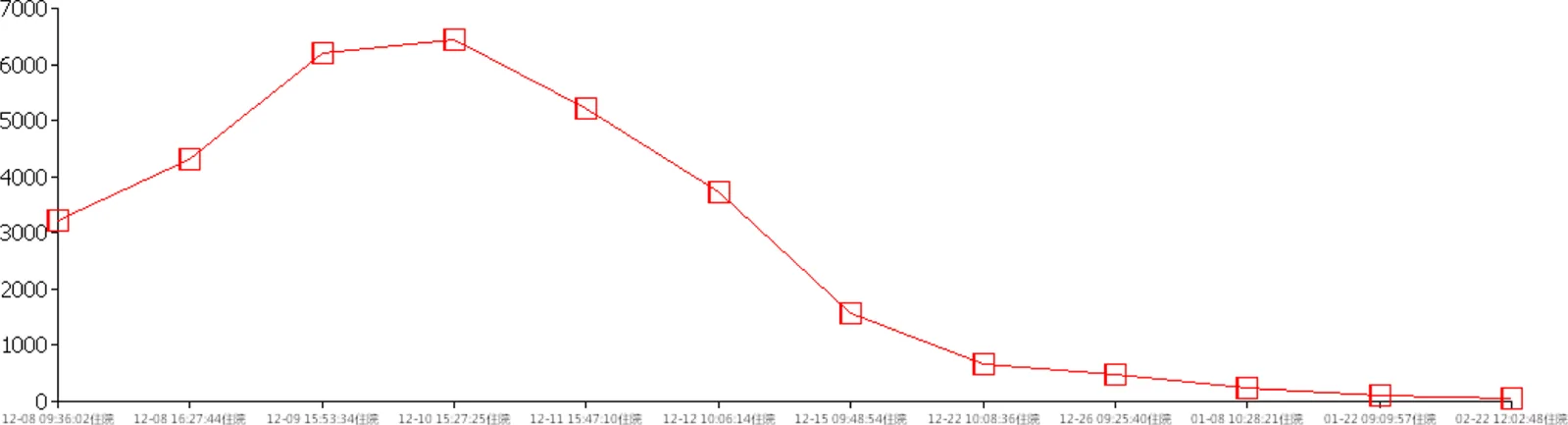
The level of serum ferritin steadily reduced to normal. When admitted, his serum ferritin (SF) (CLIA) was 3215.1 ng/mL; this graph shows a gradually decreasing trend after treatment. Bone marrow revealed 1.5% atypical lymphocytes. Hemophagocytic cells are common, with visible phagocytosis of red blood cells and platelets. *X*-axis: daytime, *Y*-axis: serum ferritin concentration. CLIA = chemiluminescence immunoassay.

Cytokines showed elevated interferon-α at 245.22 pg/mL and interferon-γ at 28.48 pg/mL, and anti-EBV capsid antigen IgG antibody was positive at 6.35. Antinuclear antibodies showed homogeneous at 1:1000, granular at 1:320, and cytoplasmic granular at 1:100. The antinuclear antibody extracted nuclear antigens spectrum revealed that the anti-double-stranded DNA antibody and anti-histone antibody were positive. Flow cytometry absolute counts of 5 subgroups showed all decreased total T cell percentage (CD3+) at 59.7%, total T cell count (CD3+) at 124 cells/μ L, a Th cell percentage (CD4+ CD3+) of 17.3%, a Th cell count (CD4+ CD3+) of 36 cells/μL, a Ts cell percentage (CD8+ CD3+) of 33.7%, a Ts cell count (CD8+ CD3+) of 70 cells/μL, a Th/Ts ratio (CD4+ CD3+/CD8+ CD3+) of 0.51, a B cell percentage (CD19+ CD3−) of 11.4%, a B cell count (CD19+ CD3−) of 23 cells/μL, and a NK cell percentage (CD16+ CD56+ CD3−) of 16.2%. NK cell count (CD16+ CD56+ CD3−) 33 cells/μL, CD45+ lymphocyte count 206 cells/μL. Decreased C3 and C4 were 0.444 and 0.080 g/L respectively, and elevated sCD25 was 1801.71 pg/mL. NK cell activity was 10.25%. Total β-human chorionic gonadotropin was <0.2 IU/L. Serum ferritin (SF) (chemiluminescence immunoassay) was 3215.1 ng/mL, showing a gradually decreasing trend (Fig. 3). Respiratory pathogen antibody spectrum and anticardiolipin antibody were all negative.

**Figure 4.**
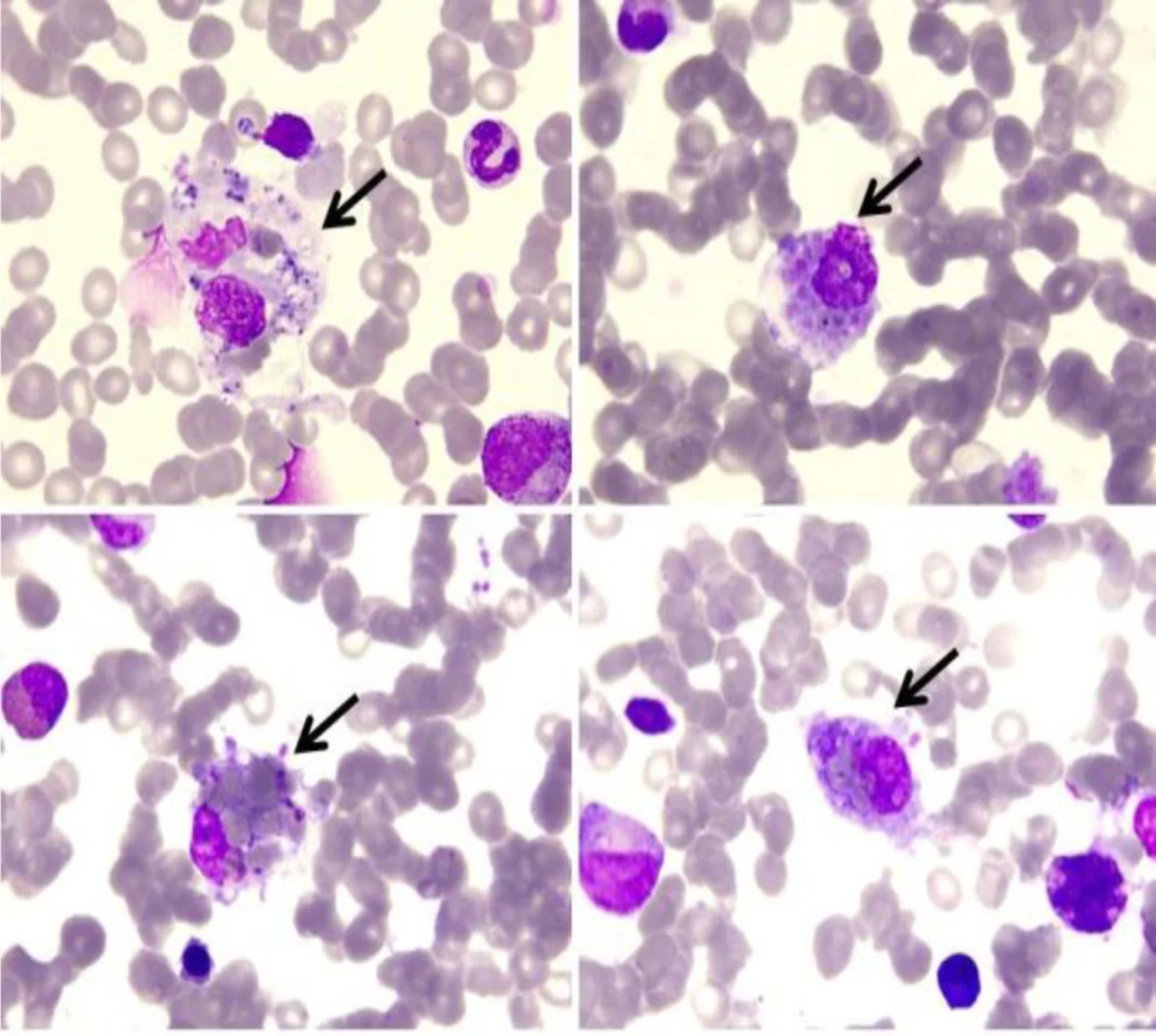
Bone marrow cytology (Wright-Giemsa stain ×1000). Hemophagocytes containing a number of mature red blood cells and platelets were observed by bone marrow cytology examination, with smaller size and irregular shape (black arrow marked).

Bone marrow examination revealed 1.5% atypical lymphocytes. Hemophagocytic cells were common, with visible phagocytosis of red blood cells and platelets. The diagnostic opinion is hemophagocytic syndrome (Fig. 4). Bone marrow pathology showed that hematopoietic tissue proliferation was reduced (about 40%–50%), while megakaryocytes, T lymphocytes, B lymphocytes, and plasma cells were scattered. Hemophilia was not obvious under HE staining, while CD163 was ++ positive and EBER of in situ hybridization was negative.

ALL bacterial cultures were negative. EB virus DNA was at 1.01E+03 copies/mL, and cytomegalovirus, influenza A virus RNA, influenza B virus RNA, COVID-19 nucleic acid, and mycoplasma pneumoniae antibodies were negative.

CT showed no significant abnormalities, ruling out acute pancreatitis.

The child was diagnosed with SLE (lupus crisis, significant involvement of the blood system, MAS, lupus nephritis, upper respiratory tract infection, abdominal pain, and EB virus infection) and treated with ceftriaxone, oseltamivir, intravenous fluid replacement, methylprednisolone, omeprazole, cyclosporine, and high-dose immunoglobulin (Table 1). On December 8th and 9th, his white blood cells decreased to 0.90 × 10^9^/L and 0.48 × 10^9^/L, and complete blood cells count showed a progressive pancytopenia. SF and lactate dehydrogenase were significantly increased. The patient was aggravated and the systemic lupus erythematosus disease activity index (SLEDAI-2000) activity was evaluated as 9 scores. From December 9th, the child was improved significantly, CBC began to gradually increase and lactate dehydrogenase, alanine aminotransferase, and aspartate aminotransferase showed a decreasing trend. The lupus crisis was relieved. On December 12th, MAS achieved partial response, and the SLEDAI-2000 activity evaluation was rated at 6 scores again. The facial erythema had subsided, and the liver had shrunk; therefore, on December 27th, the patient was discharged and regularly followed up.

**Table 1:** Exact doses/routes/durations of therapies for the patient.

Day admission	Administration
Day 2	Oseltamivir 75 mg × 5 PO, ceftriaxone 1.5 g IV × 7, methylprednisolone 48 mg/d IV × 3
Day 4	Cyclosporine 100 mg 2/d PO × 16
Day 5	Methylprednisolone 900 mg/d IV × 2
Day 7	IVIG 500 mg/kg IV × 4
Day 19	Belimumab 450 mg IV-VP × 1, prednisone acetate 60 mg/d PO × 1

## 3. Discussion

The pathogenesis of secondary MAS in SLE is not yet clear, which may be related to dysfunction of self-reactive lymphocytes and macrophages associated with lupus caused by infection.^[3]^ The excessive activation of myeloid cells of bone marrow caused by immune complexes, impaired NK cell toxicity, abnormal activation and proliferation of CD8+ T lymphocytes and monocytes/macrophages, and excessive secretion of pro-inflammatory cytokines may ultimately induce a systemic immune inflammatory response and multi-organ damage, which may be related to secondary MAS in SLE. In addition, viral infection may be another important factor for secondary MAS in SLE. The study of Gavin group showed that EBV is the most common infection inducing factor, followed by CMV infection, both of which are more common in MAS induced by SLE patients.^[4,5]^ Anti-EBV capsid antigen IgG antibodies of our patient are positive, and a copy number of the EB virus DNA test is 1.01E+03/mL, and bone marrow examination revealed 1.5% atypical lymphocytes, so EBV infection may be a contributing factor to the onset of systemic juvenile idiopathic arthritis (SJIA)-MAS. Unlike prior cases that showed high EBV loads with positive EBER, this case had low PCR and negative EBER, indicating her possible early stage of EBV infection or incorrect specimen collection.

According to the HLH-2004 diagnostic criteria,^[6]^ our patient met all 6 indicators, including fever, cytopenia, hemophagocytosis in the bone marrow, decreased or absent NK cell activity (10.25%, supportive), fibrinogen low at 1.7 g/L (supportive), increased SF peaked at 6453.5 ng/mL (HLH-2004 suggestive ≥500 μg/mL) with a gradually decreasing trend (Fig. 3), and elevated soluble interleukin-2 receptor (sCD25) at 1801.71 pg/mL (consistent with HLH); thus, the diagnosis of HLH can be reached. Based on the 2019 SLE scoring criteria,^[5]^ our child exhibited fever, leukopenia, thrombocytopenia, subacute cutaneous lupus or discoid lupus, decreased complement C3 and C4, and anti-double-stranded DNA antibodies. His total score was 23 > 10, and a definitive SLE diagnosis with agranulocytosis and lupus crisis can be made. For patients with SLE complicated by MAS, their diagnosis should meet the HLH-2004 diagnostic criteria,^[6]^ the 2016 SJIA–MAS classification criteria,^[7]^ the 2019 MAS score, and the HScore scoring system. Firstly, according to the 2016 SJIA–MAS classification criteria,^[7]^ our patient had persistent fever with consistently SF > 684 μg/mL, and his platelet count and AST were 95.0 × 10^9^/L (SJIA–MAS suggestive ≤181 × 10^9^/L) and 79.0 U/L (SJIA–MAS suggestive>48 U/L), respectively; the diagnosis of SLE complicated by MAS can be reached. Secondly, according to the 2019 MAS scoring formula, his score is 1.79 ≥ −2.1. Thirdly, according to the HScore scoring system, his score is 169 ≥ 169, indicating SLE-associated MAS. In addition, SLE-associated MAS requires differential diagnosis as follows. Firstly, sepsis, in which the primary infection is usually observed, and symptoms include fever, chills, elevated white blood cell count, and pathogen detection. Secondly, the SLE activity phase, where the SLEDAI and systemic lupus erythematosus disease activity score ^[8]^ are helpful for identification between SLE activity and infection. MAS patients exhibit elevated interleukin-2 receptor α and soluble CD163 levels; bone marrow pathology and histochemistry of our patient showed his CD163 was “++” positive. Additionally, the 2016 SJIA–MAS classification criteria^[7]^ also are helpful for identification between SLE activity and infection.

Lupus crisis can affect the kidneys, cardiovascular system, central nervous system, respiratory system, and digestive system. Our patient presented with abdominal pain symptoms, but the results of the β-human chorionic gonadotropin examination and CT ruled out pregnancy or gynecological diseases, acute appendicitis, acute gastroenteritis, and pancreatitis. Therefore, it may be related to the involvement of a lupus crisis in the digestive system. SLE involvement in the blood system can include severe hemolytic anemia, thrombocytopenic purpura, and granulocytopenia. Autoimmune hemolytic anemia (AIHA) is a manifestation of SLE recurrence, most of which have elevated reticulocytes and a positive Coombs test. Thrombocytopenia in SLE patients is associated with platelet antibodies, antiphospholipid antibodies, and impaired maturation of bone marrow megakaryocytes.^[9]^ AIHA accompanied by thrombocytopenia is called Evans syndrome (ES), which is a rare autoimmune disease composed of at least 2 autoimmune conditions: thrombocytopenia occurring simultaneously or sequentially. Autoimmune conditions includes immune thrombocytopenia, AIHA, and autoimmune neutropenia. Secondary ES is commonly seen in lymphoproliferative diseases, solid tumors/ovarian epithelial cysts/cancers, autoimmune diseases, viral infections, and primary immunodeficiency diseases. SLE is the most common autoimmune disease associated with ES.^[10]^ Our patient also mainly presents with hematological involvement. His direct Coombs test is positive for C3, indicating complement system activation and cold antibody hemolysis. Although his anticardiolipin antibodies were negative, SLE-related secondary Evans syndrome cannot be ruled out.

Our patient was treated according to the guidelines for children with lupus crises complicated with MAS, with multiple system involvement and critical condition. According to the Chinese Guidelines for the Diagnosis and Treatment of Systemic Lupus Erythematosus (2025 Edition),^[11]^ the Diagnostic and Treatment Standards for Systemic Lupus Erythematosus,^[12]^ and the Expert Consensus on Diagnosis and Treatment of Rheumatoid Disease Associated Macrophage Activation Syndrome in Children – Systemic Lupus Erythematosus in Children,^[13]^ hormone (methylprednisolone) shock combined with immunosuppressant (cyclosporine A) and high-dose immunoglobulin was administered. On December 27th, the patient’s facial butterfly-shaped erythema significantly subsided. Except for mild anemia, various indicators returned to normal range. Therefore, the patient was discharged and regularly followed up. Given that cytokine storms can lead to multiple organ failure, timely diagnosis and treatment of MAS are crucial. Therefore, when adolescent SLE patients experience unexplained fever, pancytopenia, and methemoglobinemia, MAS should be considered in a timely manner.

## 4. Conclusions

EBV induced Evans syndrome in SLE-related MAS is rare and is often misdiagnosed as HLH. The disease history, laboratory tests, and differential diagnosis are helpful for the confirmation of this disease.

## Acknowledgments

The authors express their gratitude to the patient who made this work possible, as well as the professionals and doctors that participated in therapy and the study.

## Author contributions

**Conceptualization:** Yini Chen.

**Data curation:** Ya Chen.

**Investigation:** Ya Chen.

**Methodology:** Yini Chen.

**Project administration:** Meng Zhao.

**Resources:** Yini Chen.

**Software:** Yini Chen.

**Supervision:** Shenghang Zhang.

**Visualization:** Shenghang Zhang.

**Writing – review & editing:** WeiBin Li.
